# Nectar Secretion of Floral Buds of *Tococa guianensis* Mediates Interactions With Generalist Ants That Reduce Florivory

**DOI:** 10.3389/fpls.2020.00627

**Published:** 2020-05-21

**Authors:** José Neiva Mesquita-Neto, Elder Antônio Sousa Paiva, Leonardo Galetto, Clemens Schlindwein

**Affiliations:** ^1^Departamento de Botânica, Instituto de Ciências Biológicas, Universidade Federal de Minas Gerais, Belo Horizonte, Brazil; ^2^Centro de Investigación en Estudios Avanzados del Maule, Vicerrectoría de Investigación y Postgrado, Universidad Católica del Maule, Talca, Chile; ^3^Instituto Multidisciplinario de Biología Vegetal (UNC-CONICET), Universidad Nacional de Córdoba, Córdoba, Argentina

**Keywords:** plant–herbivore interactions, domatia, florivory, myrmecophily, extrafloral nectar, floral nectar, nectaries

## Abstract

The specialised mutualism between *Tococa guianensis* and ants housed in its leaf domatia is a well-known example of myrmecophily. A pollination study on this species revealed that flowers in the bud stage exude a sugary solution that is collected by ants. Given the presence of this unexpected nectar secretion, we investigated how, where, and when floral buds of *T. guianensis* secret nectar and what function it serves. We studied a population of *T. guianensis* occurring in a swampy area in the Cerrado of Brazil by analyzing the chemical composition and secretion dynamics of the floral-bud nectar and the distribution and ultrastructure of secretory tissues. We also measured flower damage using ant-exclusion experiments. Floral bud nectar was secreted at the tip of the petals, which lack a typical glandular structure but possess distinctive mesophyll due to the presence of numerous calcium oxalate crystals. The nectar, the production of which ceased after flower opening, was composed mainly of sucrose and low amounts of glucose and fructose. Nectar was consumed by generalist ants and sporadically by stingless bees. Ant exclusion experiments resulted in significantly increased flower damage. The floral nectar of *T. guianensis* is produced during the bud stage. This bud-nectar has the extranuptial function of attracting generalist ants that reduce florivory. Pollen is the unique floral resource attracting pollinators during anthesis. *Tococa guianensis*, thus, establishes relationships with two functional groups of ant species: specialist ants acting against herbivory and generalist ants acting against florivory.

## Introduction

Nectar is a sugary secretion that mediates interactions between plants and different groups of animals (Delpino, 1886). Nectar consists predominantly of water, sugars, and a wide range of amino acids (Percival, 1961; Baker and Baker, 1973). The concentration and composition of carbohydrates and amino acids in nectar directly influence the types of visitors and their activity (Heil, 2011; Del-Claro et al., 2016).

Nectar and nectaries can be distinguished according to their location in the plant (floral and extrafloral nectar/nectaries) and their function (nuptial and extranuptial nectaries, *sensu* Delpino, 1886). Floral nectar is produced and secreted by nectaries located in different floral whorls (e.g., calyx, corolla, androecium, and gynoecium) while extrafloral nectar is produced by nectaries situated outside the flower on virtually any vegetative structure (Elias, 1972; Bentley, 1977; Dalvi et al., 2017). Nectar, from a functional point of view, has two main roles: attracting pollinators or attracting defenders (Heil, 2011). Pollinators are linked to plant reproduction when collecting nectar (nuptial function), while defenders usually discourage herbivorous insects from feeding on the plant when they are patrolling plants and consuming extranuptial nectar (Delpino, 1886 *apud* Bentley, 1977). These conceptualizations for nectaries and nectar according to their location and function may overlap in the literature. The site of nectar secretion and its function usually coincide, with most floral nectaries presenting nuptial functions and extrafloral nectaries the extranuptial function (Feldhofen, 1933). Nevertheless, this is not a rule, and sometimes extrafloral nectaries are involved in pollination (Faegri and Van Der Pijl, 1979; Weberling, 1981), while floral nectaries may act in plant defense (Paiva, 2011 and references therein; Del-Claro et al., 2013).

Extranuptial nectar, in most cases, attracts ants, most species of which have aggressive and predatory behaviors that defend the plant against herbivores (Galetto and Bernardello, 2003; Heil, 2008, 2015; Del-Claro et al., 2016). This nectar can be a critical food resource for nectarivorous ants (Davidson, 1997; Blüthgen et al., 2000; Blüthgen and Fiedler, 2004; Schmid et al., 2010), by contributing to colony growth. For some ant species, nectar can comprise up to 90% of all the food collected (Lach et al., 2009; Byk and Del-Claro, 2011), but usually accounts for up to 10% of the food requirements of a colony (Young and Hermann, 1980; Fewell et al., 1992; Tillberg and Breed, 2004). Ants generally prefer sucrose-rich nectars (Stpiczynska, 2003; Blüthgen and Fiedler, 2004; González-Teuber and Heil, 2009; Nepi et al., 2009), but a few species are not able to digest disaccharides and prefer nectars rich in hexoses (Martínez del Rio, 1990; Heil et al., 2005).

The degree of specialization of interactions between extranuptial nectars and ants can vary widely. In generalised interactions, ants usually do not live on the plant itself, and extranuptial nectar is the only food reward produced by the plant (Galetto and Bernardello, 2003; Del-Claro et al., 2013; Marazzi et al., 2013), forcing ants to complement their nutritional requirements through other food items (Heil et al., 1998; Tillberg and Breed, 2004; Feldhaar et al., 2010; Lanan and Bronstein, 2013). In these cases, plant-ant mutualisms can be opportunistic and, as a result, the quality and quantity of defense ants provide to the plant can be variable (Ness et al., 2006; Miller, 2007; Palmer et al., 2010; Del-Claro et al., 2013). Myrmecophytic mutualisms, on the other hand, are classic examples of obligatory and specialised mutualisms (Davidson and Mckey, 1993; Heil and McKey, 2003). The involved plants maintain intimate associations with ant colonies of certain species, which are housed in specific chambers, such as stems, empty spines, or domatia (Davidson and Mckey, 1993). Myrmecophytic plants (e.g., *Acacia*, *Cecropia*, *Leonardoxa*, *Piper*, *Macaranga*, and *Tococa*) generally provide all the nutrient needs of resident ants (Davidson and Mckey, 1993; Heil et al., 2004; Webber et al., 2007).

The myrmecophytic ant-domatia interaction of *Tococa guianensis* is a well-known example of obligatory and specialised mutualism (Davidson and Mckey, 1993) because the plants provide all the food needs of resident ants, and the ants protect the plant against herbivore attack (Davidson and Mckey, 1993; Heil et al., 1998). However, during a previous study on pollination and pollinators (Mesquita-Neto et al., 2018), we noted that flower buds of *T. guianensis* exude a sugary solution that attracts ants. The flowers of *T. guianensis* are visited exclusively by pollen-collecting bees (Ranieri et al., 2013; Mesquita-Neto et al., 2018). Given this unexpected and unknown floral nectar secretion, we investigated how, where, and when floral buds of *T. guianensis* secrete nectar and what function it serves. We expected that the nectar of *T. guianensis* secreted in the bud-stage be related to the attraction of flower defenders (extranuptial function). Thus, to understand the function of this nectar secretion of floral buds, we asked the following questions: (1) What is the chemical composition of this floral bud nectar, and when is it secreted? (2) What are the secretory structures, and where are they located? (3) What are the species of visiting ants? and (4) Does the presence of visiting ants reduce flower damage?

## Materials and Methods

### Species and Habitat

*Tococa guianensis* is a 1–5 m-tall shrub distributed widely in the Neotropics, where it occurs from southern Mexico to Brazil (Michelangeli, 2005). The species occurs in the Cerrado in Brazil as well as other Neotropical savannahs, especially on wet soils (Michelangeli, 2003). The flowers have poricidal anthers and are pollen-only flowers, like most other species of Melastomataceae (Buchmann, 1983).

The studied population of *T. guianensis* occurs in a swampy area close to the edges of gallery forest in *Parque Estadual do Rio Preto* (Rio Preto Nature Reserve), located in the Espinhaço Mountain Range in the state of Minas Gerais, Brazil (18°05′28.3″2S, 43°20′29.2″2W). The Park encompasses an area of about 12,000 ha, which is mainly covered with natural Cerrado vegetation (Brazilian savannah). In the study area, the shrubs flower from June to November (Ranieri et al., 2013; Mesquita-Neto et al., 2018). Field experiments and observations were carried out from September to December of 2015 and 2016. The climate of the region is tropical with well-delimited dry (April–September) and rainy (October–March) seasons; the average annual temperature is 19°C (Salino et al., 2013).

### Distribution and Structure of Nectaries

We randomly selected and bagged floral buds (*n* = 6) and open flowers (*n* = 7) with organza bags shortly before sunset (∼18:00) to exclude visitors and allow nectar to accumulate. The following morning, we collected the flowers at anthesis and floral buds with accumulated nectar and fixed them in Karnovsky solution, at pH 7.2 with 0.1 M phosphate buffer (Karnovsky, 1965), for 24 h. We then dehydrated the samples in an ethanol series and embedded them in synthetic resin (2-hydroxyethyl methacrylate) (Leica^®^), according to Paiva et al. (2011). To locate and analyze the anatomy of the nectar secreting structures, we obtained longitudinal and transverse sections (5 μm thick) with a Zeiss Hyrax M40 rotary microtome, stained them with toluidine blue solution at pH 7.4 (O’Brien et al., 1964), arranged them on slides and mounted them in Entellan^®^ for study by light microscopy. We then employed histochemical tests on resin embedded sections obtained with a rotary microtome. We used lugol for the identification of starch (Johansen, 1940), 0.02% aqueous solution of Ruthenium red for the detection of pectic compounds (Jensen, 1962), and 10% aqueous ferric chloride solution for phenolic substances (Johansen, 1940). We obtained images using an Olympus digital camera (Olympus LC20, Münster, Germany) coupled to a light microscope (Olympus CX-41, Tokyo, Japan).

For scanning electron microscopy (SEM), we fixed floral buds using Karnovsky fixative (pH 7.2 in 0.1M phosphate buffer; modified from Karnovsky, 1965) and dehydrated them in an ethanol series as described for light microscopy. The samples were critical-point dried using liquid CO_2_; glued on metallic supports for frontal, ventral, and lateral views, using carbon tape; coated with gold (Robards, 1978; Sõber et al., 2010) and observed using a Quanta 200 scanning electron microscope (FEI Company, Eindhoven, Netherlands), at 12–0 kV.

#### Transmission Electron Microscopy

For transmission electron microscopy, we subjected fragments of the distal border (2 mm long) of petals from pre-anthesis floral buds to vacuum in Karnovsky solution at pH 7.2 with 0.1M phosphate buffer (Karnovsky, 1965), fixed them for 24 h and post-fixed them in 1% osmium tetroxide (0.1M phosphate buffer, pH 7.2). We then washed the samples in phosphate buffer (0.1M, pH 7.2), dehydrated them in an ethanol series, and embedded them in low-viscosity epoxy resin (Spurr, 1969). We contrasted 50 nm ultrathin sections with uranyl acetate and lead citrate and examined them using a Tecnai G2-12-Spirit transmission electron microscope (Philips/FEI Company, Eindhoven, Netherlands) at 80 kV.

### Nectar Secretion Dynamics and Nectar Sugar Composition

We marked and bagged forty floral buds of 10 individuals of *T. guianensis* (*n* = 4 per individual) with organza bags to prevent visitor access and to allow nectar to accumulate. The buds were randomly assigned to treatments, independently of their size or developmental stage. We collected accumulated nectar at 4-h intervals during 24 h (at 20:00 h, 00:00 h, 04:00 h, 08:00 h, 12:00 h, and 16:00 h). The sampling occurred in 1 day, and all the 40 buds were sampled during every interval. We collected nectar droplets with graduated glass microcapillaries and measured nectar volume (μL) and sugar concentration (percentage of mass sugar/total mass solution) with a pocket refractometer (0–50%, wt/wt; Atago, Tokyo, Japan) at every interval. We fitted penalized quasi-likelihood (PQL) generalised mixed effects models (GLMMs) with a quasi-poisson distribution to compare differences in accumulated nectar volume (μL) among time intervals (fixed effect) using the function glmmPQL of the R package MASS. We randomly assigned plants to treatments, and so plant individual was introduced as a random effect. The volume of nectar per time interval was nested within individual plants to reflect the repeated measures of our sampling design.

To analyze sugar composition we collected accumulated drops of nectar from 10 buds of 10 random plants, which were previously bagged for at least 12 h, with graduated glass microcapillaries. We then transferred the drops to filter paper and placed them in individual sealed-tubes containing silica gel for dehydration and to prevent oxidation (Galetto and Bernardello, 2005). The stored nectar was dissolved in distilled water before sugar composition analysis by spectrophotometry. We determined absorbance at a wavelength of 340 nm using a spectrophotometer (Metrolab 330, Switzerland). For quantitative analysis, we used reagent kits for glucose, fructose, and sucrose (Sigma-Aldrich Co., St. Louis, MI, United States), following the methodologies proposed by Tölke et al. (2018) and references therein. We used a linear mixed-effects model (LMM) to compare the proportions of sugars that make up the nectar samples of the floral buds of *T. guianensis* using the lmer function of the R package lme4. We used plant individual as a random effect and sugars as fixed effects.

### Insects Associated With Floral Bud Nectar

Qualitative data related to floral bud nectar-exploring insects, such as species identity and behavior, were taken from field observations for two consecutive years (September to December 2015 and 2016). The collected insects are housed in the Entomological Collection of Universidade Federal de Minas Gerais (Centro de Coleções Taxonômicas da UFMG, Belo Horizonte, Brazil). To relate quantitative ant data to nectar secretion pattern during a period of 1 day, we collected all ants and other insects from the floral buds of the 10 tagged individuals of *T. guianensis* (10 min per plant) at regular 4-h intervals for 24 h (at 20:00 h, 00:00 h, 04:00 h, 08:00 h, 12:00 h and 16:00 h). The sampling occurred over a total of 10 h (1.7 h per interval^∗^6 intervals) on 1 day. We tagged the sampled ants with the number of the individual plant and collection time. We then identified the ants to species level in the laboratory. Thus, we determined the spectrum of ant visitors, as well as their relative abundance, in floral buds throughout day and night.

#### Ant Exclusion Experiment and Floral Damage

We experimentally excluded ants from *T. guianensis* plants to compare damage to flowers of these ant-excluded plants with those of plants visited by ants (control). For the treatment group (*n* = 10 ant-excluded shrubs), we applied a sticky resin (Tanglefoot^TM^) at the base of the trunk and inflorescences to hamper the traffic of ants and other crawling insects on the plant. After the application of the resin, we scanned the surface of the shrubs and removed any remaining insects manually. We also established a control group of 10 individual plants on which ants had free access to floral buds. We assigned five inflorescences of 10 individuals to each group (treatment and control) and counted the total number of floral buds per inflorescence. We counted the number of intact and damaged buds or flowers after 48 h. Floral bud damage was considered any eaten part of the corolla or holes in, or complete destruction of buds. We obtained the proportion of damaged buds as the ratio between the total initial number of buds and the number of buds damaged on each plant 48 h later. We used a GLMM (Gaussian distribution) to compare the proportion of florivory between control (plant individuals with ants) and treatment (ant-excluded plants) groups using the glmmPQL function of the R package MASS. Plant individual was included as a random effect.

## Results

### Floral Morphology and Nectar-Producing Tissues

**FIGURE 1:**
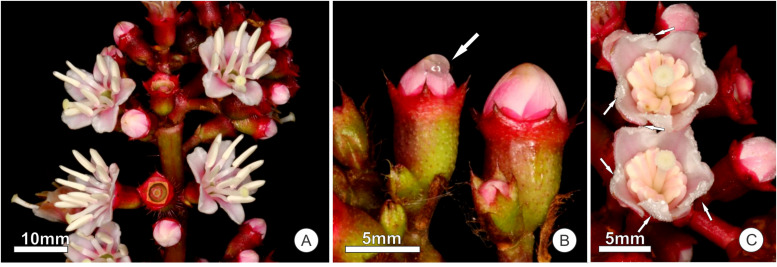
Floral structures of *Tococa guianensis*. **(A)** Inflorescence with floral buds and flowers at anthesis; **(B)** Floral buds of a previously bagged inflorescence (not accessible to visitors), one with a drop of accumulated nectar (arrow); **(C)** Freshly opened flowers with still folded stamens of a previously bagged inflorescence showing secretion droplets at the tip of the petals (arrows).

The inflorescence of *T. guianensis* is a cymous dichasia with short-pedicellate flowers (Figure 1A). The pentamerous corolla has light pink to white petals, which are 6–8 mm long, 4–5 mm wide, smooth and velvety. The androecium is the most prominent whorl, with 10 equally sized stamens with white 5 mm-long anthers (Figure 1A). At the bud stage, the contorted corolla exudes a conspicuous drop of floral nectar at the tip (Figure 1B). This secretory stage extends from young buds to the beginning of anthesis, that is, a few minutes before floral opening. During the first hour of anthesis, secretion could be seen emerging from the apex of the petal (Figure 1C). Secretion ceased when the corolla fully opened; the floral lifespan lasted up to 24 h.

**FIGURE 2:**
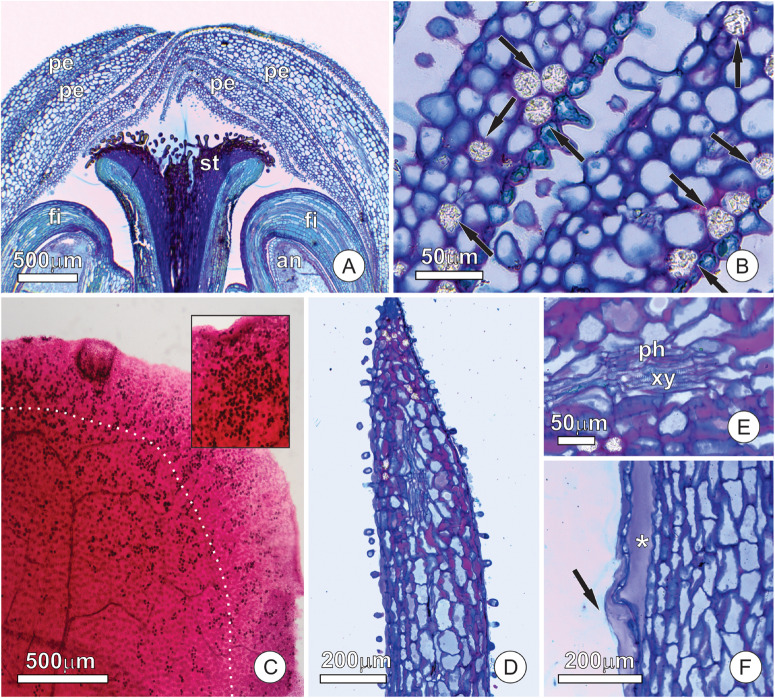
Distribution of tissues and structures involved in nectar secretion at the apex of the floral buds of *Tococa guianensis*. **(A,B)** Uniform and papillose epidermis of adaxial and abaxial petal surfaces. **(A)** Apex of floral bud in longitudinal section; **(B)** mesophyll of petals composed of spongy parenchyma with globe-shaped cells and large vacuoles containing phenolic substances and large calcium oxalate crystals (arrows); **(C)** Clarified leaf stained with safranin showing calcium oxalate crystals (dark points); notice that these crystals are strongly concentrated in an area of about 0.5 mm wide at the border of the petal (see limit by a dotted line). The insert shows how concentrated the crystals can appear; **(D–F)** apical portion of a petal **(E)** showing thick cell walls in red due to the presence of pectin and vascular bundle with phloem and xylem cells; **(F)** Margin of the apical portion of the petal showing subepidermal pectin pocket (*) with released content (arrow) (an, anther, fi, filament, pe, petal, st, stigma, ph, phloem, xy, xylem).

We found no evidence of differentiated secreting tissue in the flowers of *T. guianensis*. At the site of nectar release, the petals showed no evidence of a conspicuous nectary, with both petal surfaces being covered by a uniform and papillose epidermis (Figures 2A,B). The mesophyll of the petals was homogeneous, composed of spongy parenchyma with globe-shaped cells (Figure 2B). These cells possessed a large vacuole with phenolic substances and large calcium oxalate crystals (druses) (Figure 2B), whose density increased toward the petal apex (Figure 2C). The apical portion of the petals, corresponding to the nectar releasing area, had no special vascular system, and just some collateral vascular bundles with xylem and phloem cells (Figures 2D,E). The parenchyma cells of the mesophyll had pectin-rich primary walls with large intercellular spaces, which were mainly subepidermal and full of pectin. These pectin pockets release their contents to the outside when ruptured (Figure 2F).

**FIGURE 3:**
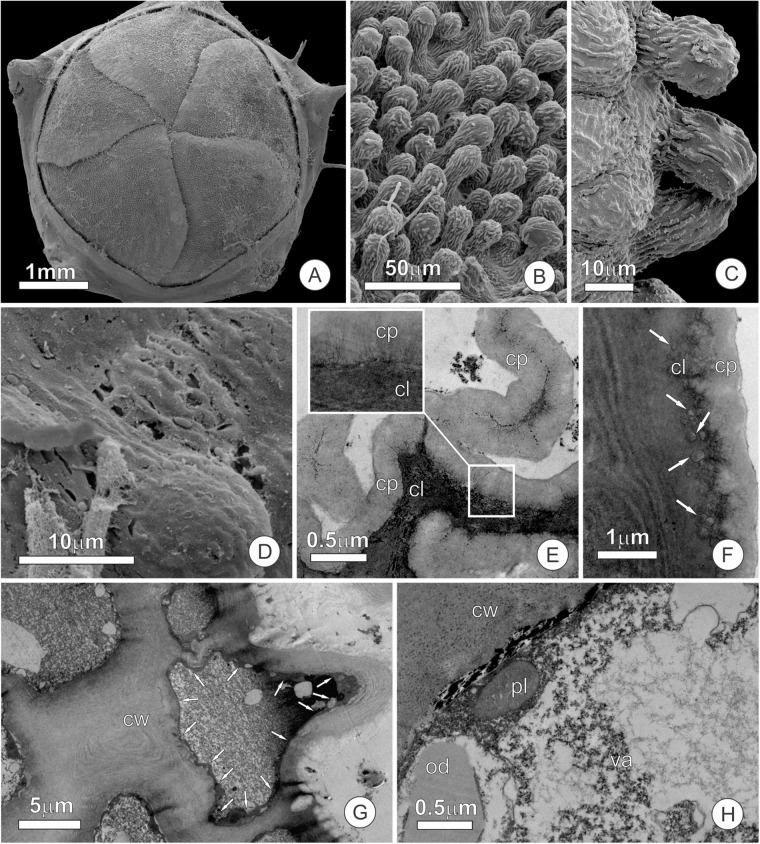
Micromorphology and ultrastructure of the apex of petals of *Tococa guianensis* in bud stage. **(A–D)** SEM images of petal surface. **(A)** Entire flower bud; note the imbricated petals. **(B,C)** Detail of abaxial surface of petals, presenting short papillae-like trichomes. **(D)** Detail of cuticle surface showing small pores. **(E–H)** Ultrastructure of cells at the distal portion of the petals. **(E,F)** Detail of cuticle in striated portion; notice cell wall elements that extend into the cuticle proper [see insert on **(E)**]. Arrows in **(F)** indicate small pockets under the cuticle proper. **(G,H)** Cytoplasm of an epidermal **(G)** and a parenchyma **(H)** cell with a large central vacuole with phenolics and scarce organelles (cl, cuticular layer, cp, cuticle proper, cw, cell wall, od, oil droplet, pl, plastid, va, vacuole).

The velvety appearance of the petal surface is due to short papillae-like trichomes (Figures 3A,B), which occur in high density and extend to the edges of the petal (Figures 3B,C). All of the epidermal cells, whether of ordinary cells or short trichomes, were non-glandular and had a thick wavy cuticle that makes the surface striate (Figure 3C). Small pores (about 1 μm) are spread over a thin film of cuticular waxes (Figure 3D), but do not extend across the cuticle proper. We observed a firmly adhered cuticle, in which wall elements extend into the cuticular layer toward the surface (Figures 3E,F). Both epidermal and parenchyma cells have organelle-poor cytoplasm with a large central vacuole (Figure 3G) where phenolic substances accumulated. This vacuole is very large and pushes the remaining portion of the cytoplasm against the cell wall (Figure 3G), where a few oil droplets and organelles, such as plastids (Figure 3H) and mitochondria, could be observed.

### Nectar Properties and Production Pattern

**FIGURE 4:**
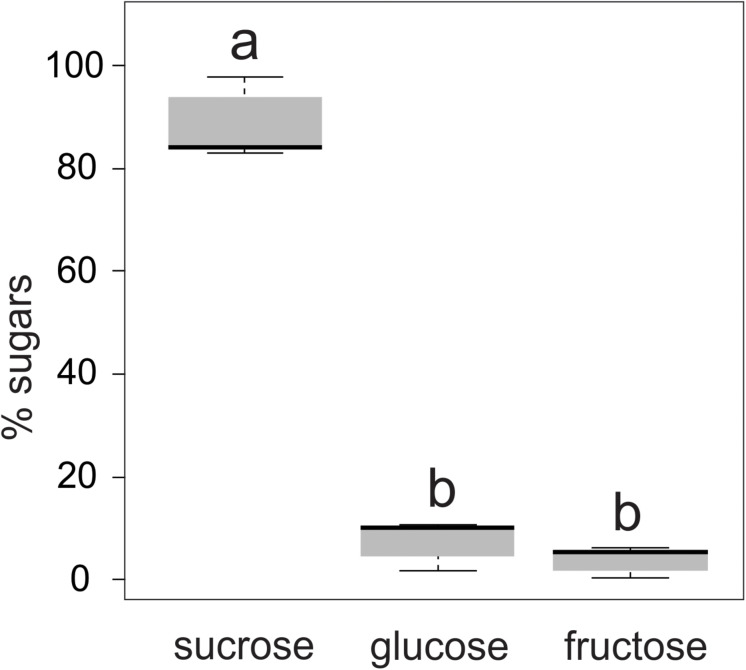
Relative proportion of sugars present in the nectar secreted by the floral buds of *Tococa guianensis* (*n* = 10 floral buds, 10 plants). Different letters indicate significant differences (LMM: χ^2^ = 1237.4, d.f. =2, *P* ≤ 0.001).

The nectar secreted by the floral buds of *T. guianensis* was colorless and had a viscous consistency. The sugar concentration of the nectar ranged from 15 to 20% (wt/wt). Sucrose accounted for 83–98% of the sugars in the nectar, while hexoses (glucose and fructose) made up the remaining 2–17% (Figure 4). The glucose content of the nectar samples was slightly, but not significantly, higher than that of fructose (LMM: χ^2^ = 1237.4, d.f. = 2, *P* ≤ 0.001, AIC = 96.4).

**FIGURE 5:**
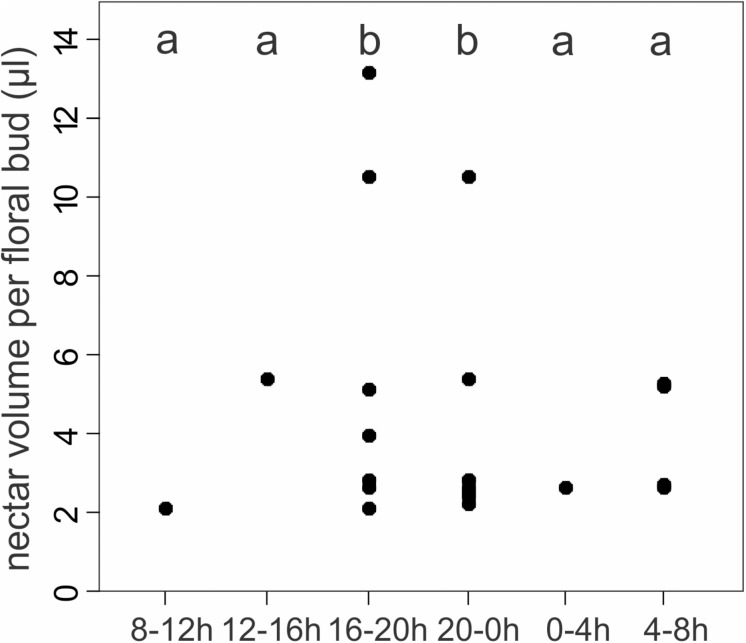
Nectar volume of floral bud secreting nectar per time interval for 41 bagged floral buds from 10 individuals of *Tococa guianensis*. Nectar is secreted in all 4-h time intervals around the clock. Different letters on topside indicate significant differences in nectar secretion (GLMM: χ^2^ = 16.9, d.f = 5, *P* ≤ 0.005, quasi-poisson distribution). Number of floral buds secreting nectar: 8–12 h: 1 bud; 12–16 h: 1 bud; 16–20 h: 7 buds; 20-0 h: 8 buds; 0–4 h: 1 bud; 4–8 h: 4 buds.

Floral buds of different sizes secreted nectar beginning in earliest bud developmental stages (<2 mm) until bud opening (>9 mm). We recorded nectar secretion in all monitored time intervals throughout the day and night (Figure 5). Only 27% of the floral buds (11) accumulated nectar in any time interval, and a given bud produced nectar only during one or two of the six four-hour intervals, with the exception of one bud (among the total number of buds labeled) that accumulated nectar in each of all sampling intervals. We noted the highest number of floral buds with accumulated nectar during early night (20–24 h; eight buds, 19.5%), while nectar accumulated in seven (17%) buds during the evening (16–20 h) (Figure 5). The volume of nectar secreted per floral bud also varied among sampling intervals (GLMM: χ^2^ = 16.9, *P* ≤ 0.005; Figure 5). There was a peak volume of accumulated nectar in the late afternoon until midnight (16–24 h; Figure 5).

### Insects Attracted to Flower Bud Nectar

**FIGURE 6:**
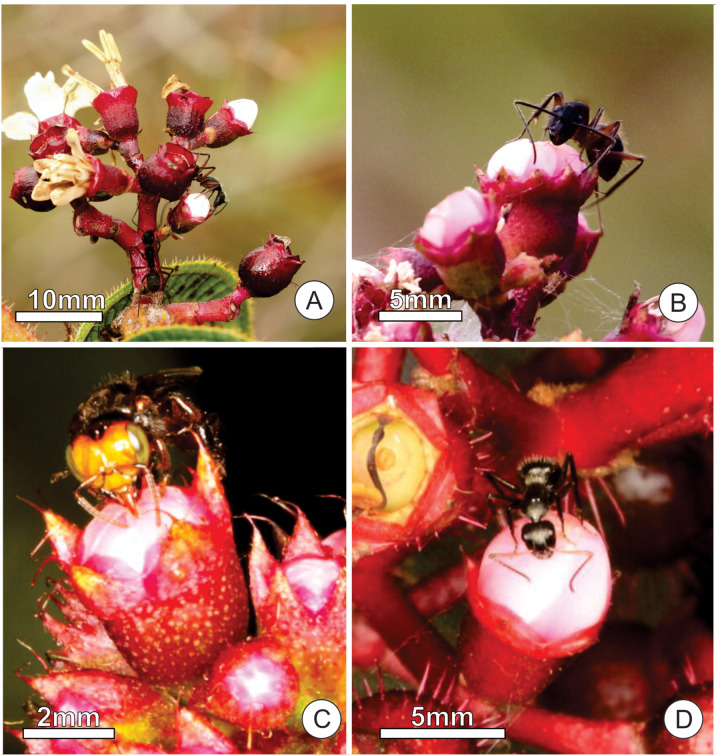
Visitors of the floral buds of *Tococa guianensis*. **(A,B)** *Camponotus rufipes*; **(C)** Worker bee of *Oxytrigona tataira*; **(D)** *Camponotus crassus*.

**FIGURE 7:**
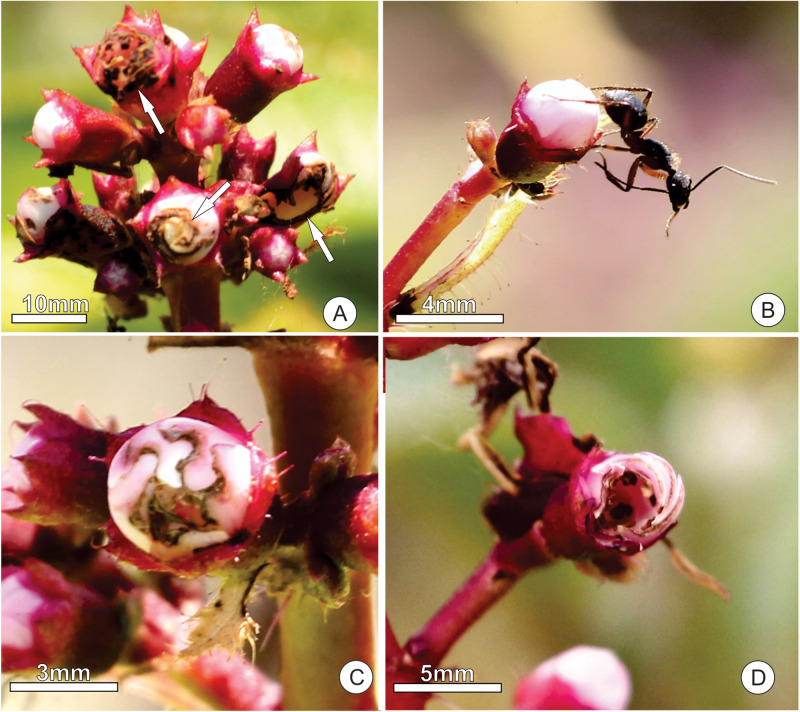
Observed damage to the floral buds and attack behavior of ants on *Tococa guianensis*. **(A)** arrows pointing to some floral damage in buds of an inflorescence; **(B)** aggressive behavior (mandible opening) of *Camponotus rufipes* on a floral bud; **(C)** floral bud with petals damaged at the apical portion; **(D)** floral bud damaged laterally, with stamens, stigma and style removed.

**FIGURE 8:**
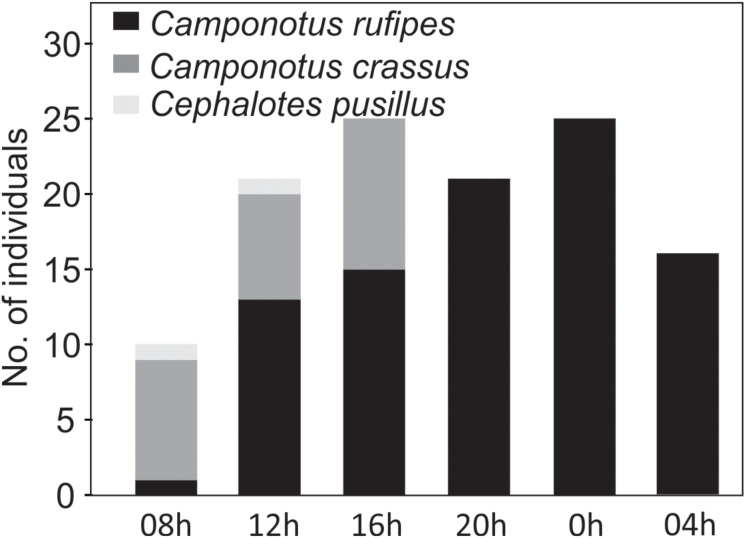
Number of individuals of the three ant species sampled visiting the 41 focal floral buds of ten plant individuals (10 min per plant; 100 min per time interval) of *Tococa guianensis* to take up nectar. *Camponotus rufipes* was recorded in all day and night intervals, while *Camponotus crassus* and *Cephalotes pusillus* visited the floral buds exclusively during daylight hours.

Nectar droplets attracted insect visitors, mainly ants and sporadically wasps and stingless bees (Figure 6). We recorded the ant species *Camponotus rufipes* (Fabricius), *Camponotus crassus* (Mayr), and *Cephalotes pusillus* (Klug), along with sporadic visits of *Oxytrigona tataira* (Smith) (Apidae, Meliponini) (Figure 6C) and an unidentified wasp. Species of the genus *Camponotus* were the most common ants seeking floral bud nectar. These ants exhibited aggressive behavior (attacks, approaches, and offensive tail-flips) toward other insects or animals when these approached floral buds (Figure 7B). However, these ants did not display aggressive behavior toward pollinators (we recorded 103 floral visits of 19 bee species). In contrast, *C. pusillus* exhibited a fugitive behavior when other insects arrived at the inflorescences. We found individuals of *C. rufipes* in floral buds during all day and night sampling intervals while those of *C. crassus* and *C. pusillus* were recorded exclusively during the day (Figure 8).

### Ant Exclusion Experiment

**FIGURE 9:**
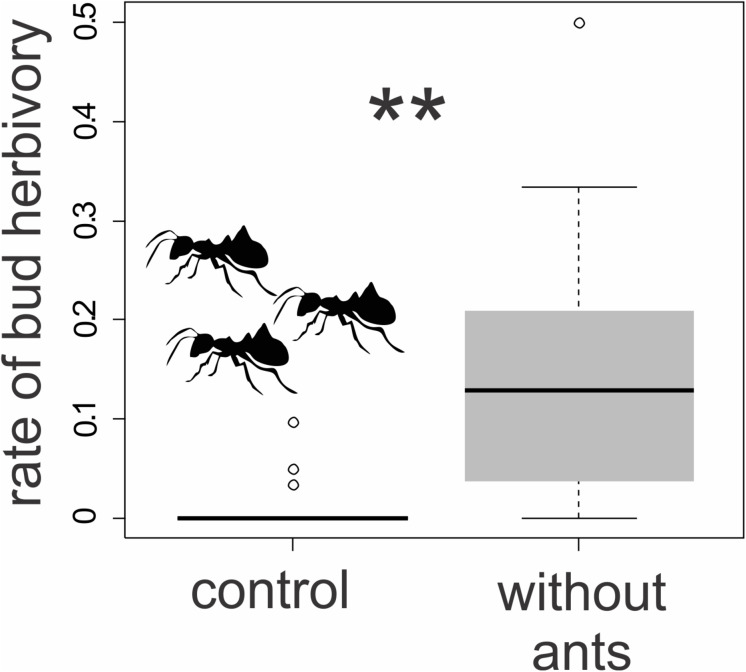
Ant exclusion experiment of *Tococa guianensis*. Proportion of damaged floral buds after 48 h in ant-excluded plants (*n* = 10 individuals; the access of ants was prevented by the application of sticky resin at the base of the trunk) and in control plants with free access for ants (*n* = 10 individuals). **Indicates statistical significance (χ^2^ = 18.45, d.f. = 1, *P* ≤ 0.001, GLMM, gaussian distribution).

We found visible damage to the floral buds of *T. guianensis*, which varied considerably from tiny holes or small removed petal parts, such as petal borders, to the removal of almost the entire bud (Figure 7). Plants with free access for ants to the floral bud nectar (control group) had a proportion of floral damage close to zero (0.07 ± 0.11). Florivory was, on average, 15 times (1500%) higher (1.07 ± 0.11; GLMM: χ^2^ = 18.45, d.f. = 1, *P* ≤ 0.001; Figure 9) in ant-excluded plants (treatment). We noted grasshoppers (adults and nymphs, Orthoptera) consuming parts of the buds of control and treated plants and larvae of *Rekora* cf. *marius* (Lycaenidae, Lepidoptera) in plants of the control group.

## Discussion

The present study shows that the nectar produced in floral buds of *T. guianensis*, before flower opening, attracts ants that protect the flowers against florivores. This nectar is not involved in pollination but rather in the attraction of flower defenders. Thus, the floral nectar has an extranuptial function because buds are visited and protected by generalist ant species commonly found on other plant species, with and without extrafloral nectaries, in the Cerrado (Belchior et al., 2016; Sendoya et al., 2016). Sucrose, which makes up the major constituent of the floral bud nectar of *T. guianensis*, seems to impose restrictions on domatia-nesting ants, as obligatory mutualistic ants usually lack enzymes able to digest this disaccharide (Heil et al., 2005; Kautz et al., 2009). Therefore, according to our findings, this interaction involves mutual benefits because *T. guianensis* buds provide food (floral nectar) to ants, and the aggressive and predatory behavior of the ants reduce damage to the floral buds. Therefore, this plant-ant relationship fits the criteria to be considered a mutualism (Bronstein, 2015).

### How and Where Is Floral Bud Nectar Secreted?

The nectar observed in the apical portion of the petals of *T. guianensis* is produced by a non-structured nectary, with no indication of cells related to its synthesis. This kind of extranuptial, non-structured floral nectary has been reported for some species of Bromeliaceae (Galetto and Bernardello, 1992). The cells of the petals, both epidermal and mesophyllous, accumulate phenolic substances and are organelle-poor, with no indication of metabolism related to the secretory process. By showing nectar secretion in wounded leaves, Heil (2016) demonstrated that few structural elements are required to produce a functional nectary.

The presence of calcium oxalate crystals in structures related to nectar secretion, as observed in *T. guianensis*, seems to be very common, as pointed-out by Gonçalves-Souza et al. (2016). The presence of calcium crystal inclusions near the vascular system may be related to the translocation of solutes through the phloem (Metcalfe and Chalk, 1979; Paiva and Machado, 2005; Pireda et al., 2018) and seems to be important for allowing phloem transport (see Paiva, 2019 and references therein). Therefore, considering the absence of nectar secreting cells, we hypothesize that phloem is the main source of the sucrose in flower bud nectar of *T. guianensis*.

Although some ruptures were observed on the cuticle surface, they were superficial and did not constitute channels. Consequently, they do not allow nectar release. Considering the absence of stomata or evidence of cuticle rupture as alternatives for the release of nectar, hydrophilic channels in the cuticle could function in nectar release. Hydrophilic bridges formed by wall elements and pectin appear to be nectar release routes, allowing passage through the cuticle, as pointed-out by Paiva (2017). Similar hydrophilic pathways crossing the cuticle have been described for stomata-free floral nectaries in Orchidaceae (Stpiczynska, 2003) and other plant families (Antón and Kamińska, 2015; Weryszko-Chmielewska and Chwil, 2016). Considering this hypothesis, nectar flow must be slow and continuous toward the petal surface because it needs to pass through countless ramifications of hydrophilic projections within the cuticular layer (Paiva, 2017). However, the nectar flow observed in *T. guianensis* is intense at certain moments, which makes the hypothesis of nectar release by epidermal ruptures quite probable. Such ruptures are caused by the accumulation of pectin in subepidermal pockets. The high viscosity of the nectar of *T. guianensis* seems consistent with this hypothesis since the sugar concentration of bud nectar was rather low and polysaccharides and nectar are released through the same rupture, mixing highly hydrophilic pectins with sugars. Thus, we consider that the high viscosity of the nectar is a consequence of the high concentration of pectins, not of sugars.

### What Is the Function of Nectar in the Bud-Stage?

Nectar secreted in flowers usually functions as a reward for pollinators (De la Barrera and Nobel, 2004; Brandenburg et al., 2009). Exceptions include floral nectaries that may play a role in attracting florivores, including ants, keeping them away from structures that are important for reproduction (Del-Claro et al., 2016; Zhu et al., 2017; Villamil et al., 2019). Our results demonstrate that ant attraction by floral bud nectar reduces florivory in *T. guianensis*, which corresponds to an extranuptial function because the floral nectar does not attract pollinators. After the unfolding of petals, the flowers are nectarless, and ants were not attracted to reproductive structures after the bud-stage.

### What Is the Chemical Composition of the Floral Bud Nectar?

Almost all the sugar that makes up the nectar of floral buds of *T. guianensis* is sucrose, a disaccharide predominant in phloem sap (Hall and Baker, 1972; Hayashi and Chino, 1990; De la Barrera and Nobel, 2004). The presence of fructose, glucose and other hexoses in nectar is the result of the activity of invertase enzymes that degrade sucrose from phloem sap into monosaccharides (Pate et al., 1985; Heil et al., 2005). Therefore, the predominance of sucrose over hexoses in the floral bud nectar of *T. guianensis* must be related to the origin of the phloem sap, with little participation of invertases.

### *Tococa guianensis*-Ant Interactions

In the present study, the floral buds of *T. guianensis* were not commonly visited by myrmecophytic ants, which usually nest in domatia (*Allomerus*, *Azteca*, and *Crematogaster*, Bizerril and Vieira, 2002; Michelangeli, 2003). The sucrose-rich nectar of the floral buds of *T. guianensis* is less attractive for some myrmecophytic ants since these ant species lack the invertases required to digest this disaccharide (Heil et al., 2005; Kautz et al., 2009; but see Galetto and Bernardello, 1992 and reference therein). Generalist ant species, however, are commonly equipped with this enzyme in their digestive tracts and have a strong preference for sucrose-rich nectars (Cornelius et al., 1996; Stapel et al., 1997; Boevé and Wäckers, 2003; Blüthgen and Fiedler, 2004; Nepi and Stpiczyńska, 2008; González-Teuber and Heil, 2009; Nepi et al., 2009). *Camponotus rufipes* and *C*. *crassus*, the most common ant species on the floral buds of *T. guianensis*, are also commonly found on other plant species in the Cerrado (Oliveira and Brandâo, 1991; Lange et al., 2013; Dáttilo et al., 2014; Ronque et al., 2018). None of the species of the genus *Camponotus* are known to be associated with any obligatory mutualism (Belchior et al., 2016; Del-Claro et al., 2016; Sendoya et al., 2016). While *C. rufipes* forages during both day and night, *C. crassus* is restricted to diurnal foraging on other plant species of the Cerrado (Oliveira and Pie, 1998; Cogni et al., 2000; Tavares et al., 2008; Stefani et al., 2015), as observed for floral buds of *T. guianensis*. The richness of sucrose in the floral bud nectar might explain why the domatia-nesting ants of *T. guianensis* avoid this nectar.

Although the symbiotic interaction between ants and the foliar domatia of species of *Tococa* has been well studied (e.g., Cabrera and Jaffé, 1994; Alvarez et al., 2001; Dejean et al., 2006; Moraes and Vasconcelos, 2009; Michelangeli, 2010), the interaction between ants and floral bud nectar in this genus was previously unknown. While specialist ants house their colonies in leaf domatia and protect the plant against non-floral herbivores, generalist ants have their colonies elsewhere (Yamamoto and Del-Claro, 2008; Weidenmüller et al., 2009; Belchior et al., 2016) and protect the plant against florivory (herbivores specialised on flowers). Here, we clarify the basic aspects of the biology of this new ant-*Tococa* interaction. Based on our findings, several new questions arise. Since *T. guianensis* has a wide area of occurrence, from southern Mexico to Brazil (Michelangeli, 2005), it would be interesting to survey whether (and which) other generalist ant species are involved in this type of relationship and whether there is variation in the pattern of nectar secretion among other populations. Also, do the generalist and aggressive ant species of *Camponotus* (Anjos et al., 2017) provide better protection against florivores than obligatory mutualistic ants? Furthermore, we expect that other species of the genus *Tococa* also maintain associations with flower defending ants because the rather inconspicuous, and thus far unknown, nectar droplets of the flower buds have probably been overlooked. Thus, this pattern of two different functional associations between ants and plants could be widespread.

We conclude that the nectar produced in the floral buds of *T. guianensis* mediates interactions with defenders. This nectar, secreted by unstructured tissue on the apical portion of the petals, is consumed by ants, which, in turn, contribute to reducing florivory. After flower opening, the production of nectar ceases, and floral visitors collect only pollen (Ranieri et al., 2013; Mesquita-Neto et al., 2018). Thus, this floral nectar, secreted at bud-stage, is not directly involved in floral resource production to pollinators but attracts generalist ants that reduce florivory.

## Data Availability Statement

The datasets generated for this study can be found in the Figshare repository doi: 10.3389/fpls.2020.00627.

## Author Contributions

JM-N, EP, and CS conceived the ideas and designed the methodology. JM-N, EP, LG, and CS collected the data. JM-N, LG, and EP analyzed the data. JM-N and CS led the writing of the manuscript. All authors contributed critically to the drafts and gave final approval for publication.

## Conflict of Interest

The authors declare that the research was conducted in the absence of any commercial or financial relationships that could be construed as a potential conflict of interest.
